# Students’ satisfaction and continued intention toward e-learning: a theory-based study

**DOI:** 10.1080/10872981.2021.1961348

**Published:** 2021-08-02

**Authors:** Mona T. Rajeh, Fahad H. Abduljabbar, Saad M. Alqahtani, Feras J. Waly, Ibrahim Alnaami, Abdulaziz Aljurayyan, Naweed Alzaman

**Affiliations:** a Dental Public Health Division, Preventative Dentistry Department, College of Dentistry, Umm Al-Qura University, Makkah, Saudi Arabia; b Department of Orthopedic Surgery, King Abdulaziz University, Jeddah, Saudi Arabia; c Department of Orthopaedic Surgery, King Fahd Hospital of the University, Imam Abdulrahman Bin Faisal University, Al-Khobar, Saudi Arabia; d Department of Surgery, Faculty of Medicine, University of Tabuk, Tabuk, Saudi Arabia; e Division of Neurosurgery, Department of Surgery, King Khalid University, Abha, Saudi Arabia; f Department of Orthopedic Surgery, College of Medicine, King Saud University, Riyadh, Saudi Arabia; g Department of Internal Medicine, College of Medicine, Taibah University, Almadinah Almunawwarah, Saudi Arabia

**Keywords:** Continuous intention, e-learning, expectation-confirmation theory, satisfaction, theory of planned behavior

## Abstract

Coronavirus disease (COVID-19) has forced the urgent lockdown of schools and colleges worldwide. To ensure the continuity of education a shift from traditional teaching to e-learning was required. This study aims to identify factors that affect students’ satisfaction and continued intention towards e-learning. A questionnaire was distributed to medical and dental students (second to sixth year) from different universities in Saudi Arabia. The study synthesizes the expectation-confirmation theory (ECT) and the theory of planned behavior (TPB) to predict students’ satisfaction and intention to continue using e-learning using a validated self-administered questionnaire. We used the structural equation model to analyze the results and assess the study’s hypotheses. A total of 870 completed questionnaires were received (67% response rate). The results showed that students were at a moderate level of satisfaction (median = 3.5). According to the ECT, both perceived usefulness and confirmation significantly influenced students’ satisfaction (β = −.69 and β = .82, respectively). Satisfaction was the strongest predictor of students’ continued intention (β = 1.95). Among the TPB constructs, perceived behavioral control (β = .51), attitudes (β = .39), and subjective norms (β = .36) had a significant positive influence on their intention to use e-learning. The results suggest efforts to increase students’ satisfaction and intention with e-learning should be directed to adopting easy and useful e-learning platforms. In addition, training and motivating students to continue e-learning and increasing their confidence to ensure the effective and efficient use of such teaching modalities.

## Introduction

At the end of 2019, COVID-19, a highly infectious disease that originated in Wuhan city, started to spread all over China [1]. In March of 2020, the World Health Organization announced the outbreak of the virus as a pandemic [2]. Social distancing was the most effective strategy to prevent the spread of the disease until a vaccine or treatment is invented. In response to the pandemic, governments in many countries considered lockdowns in schools and universities where crowds could not be avoided. In turn, this tragedy shifted the pendulum of learning exclusively from traditional face-to-face teaching to e-learning.

E-learning uses information and communication technologies to facilitate access to online teaching resources and provide students with collaborative environments [1]. The proliferation of new technologies made it practical to implement e-learning in academic institutions. Educators have successfully made many efforts to utilize the available technologies to continue the educational process during this crisis.

The sudden shift to e-learning is challenging in clinical healthcare courses requiring hands-on skills training [2]. However, medical and dental educators are working to adopt innovative learning methods, including live streaming lectures and virtual reality simulations, to motivate students to continue learning [3–5]. They used different videoconference systems, such as Blackboard, Zoom, and WebEx to deliver educational content [6,7].

Several studies reveal that e-learning is more effective than traditional learning [8,9]. This teaching strategy eliminates the time and distance barriers and improves access to education. Flexibility is another benefit of e-learning. Students can take their online courses anytime and anywhere, enabling them to become lifelong learners [10]. Nevertheless, e-learning has few drawbacks, like social isolation, lack of communication and interaction, and network problems [11].

Before the era of COVID-19, e-learning was not the most common teaching method within medical and dental schools in Saudi Arabia. Like other countries, Saudi Arabia has supported the sudden transmission to adopt this new teaching modality in the educational curriculum. Most medical and dental schools are preparing to transform their education methods from traditional to virtual classes until the pandemic is over [12,13]. As students are the future generation of doctors and dentists, it is crucial to study their opinions regarding the online learning approach. Unfortunately, there is a scarcity of knowledge on e-learning acceptance in Saudi Arabia. Therefore, this study aims to identify the factors influencing medical and dental students’ satisfaction and continued intention toward e-learning. The expectation-confirmation theory (ECT) and the theory of planned behavior (TPB) guided the study.

## Theoretical background

### Expectation-confirmation theory (ECM)

The expectation-confirmation model (ECM) is commonly used in the marketing field to study customer satisfaction and post-purchase behavior, but is rarely used in healthcare [14]. In recent years, ECM has been increasingly applied in the field of information technology such as World Wide Web to explain and predict usage continues intentions [15,16]. ECM, developed by Bhattacherjee, proposes that users’ continued intention to use information technology is theoretically dependent on their perceived usefulness, the extent of their confirmation, and their satisfaction with the use of this technology [14]. Confirmation refers to ‘the degree of users’ perception of the congruence between expectations of the technology’s usefulness and its actual performance’ [14]. Expectation (perceived usefulness) is known as ‘the degree to which a person believes that using a specific system is useful and would enhance their performance’ [17]. Spreng et al. defined satisfaction as ‘an affective state that is the emotional reaction to a product or a service experience’ [18]. Continuance intention is defined as ‘the behavior of a user to continue using a service after accepting it’ [14].

According to the theory, the consumer’s confirmation of expectations and the technology’s perceived usefulness determine the consumer’s satisfaction [14,19]. Accordingly, consumers’ satisfaction with information technology positively affects their intention to continue using it. In addition, the ECM posits that consumers’ perceived usefulness positively affects their intention to continue technology usage [14]. As e-learning is a type of information technology, previous studies indicated that ECM had successfully predicted users’ intention to continue using e-learning [19,20]. Therefore, Lee adopted the ECM to examine how students’ confirmation of e-learning impacts their satisfaction and continued intention to use e-learning [19]. Furthermore, Chuo et al. conducted a study to understand factors that lead patients to continue using e-learning technologies based on the ECM [20]. Their results revealed that e-learning continued intention is significantly related to patients’ satisfaction and confirmation of expectation [20].

### Theory of planned behavior (TPB)

Ajzen developed the TPB, which assumes that individuals’ attitudes, subjective norms and perceived behavioral control predict their intention to engage in a given behavior [21,22]. Attitudes refer to ‘the level to which an individual favors a given behavior’ [22]. Subjective norms are ‘the social pressure that influences individuals to perform a behavior’ [22]. Finally, perceived behavior control is ‘an individual’s perception of the difficulty or ease of performing a behavior’ [23].

This theory explains individuals’ behavior towards adopting new technology very well. TPB has been applied in several contexts, such as psychology, technology, and healthcare [24,25]. In medical education, several studies used TPB to study factors affecting students’ readiness to adopt mobile learning or examine general practitioners perceived barriers to using e-learning [24,26,27]. In this regard, Hadadgar et al. revealed that attitudes and perceived behavioral control are significant factors that predict general practitioners’ intention to use e-learning in continued medical education [28]. While in dentistry, Nkenke et al. used the theory to predict students’ acceptance of virtual planning software for dental implants [29]. Their results showed that students’ attitudes positively affect the intention to accept this new technology.

### Conceptual model and hypotheses

**Figure 1.**
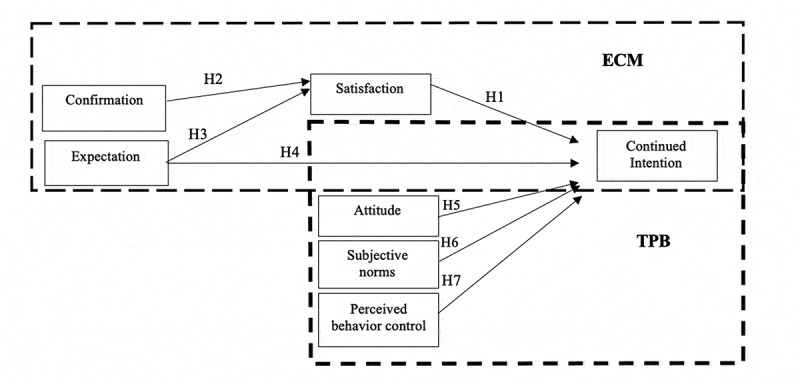
Research model and hypotheses

The conceptual model comprises seven constructs based on ECT and TPB. The operational definitions of the constructs and the hypothesized relationship between them are presented in Figure 1 and Table 1. From the available literature and grounded on Lee’s work [19] that combines ECM and TPB to predict students’ intentions to continue taking e-learning courses, this study investigated the factors influencing medical and dental students’ satisfaction and continued intention toward e-learning, guided by the integration of ECT and TPB. Based on the abovementioned theories, the hypotheses proposed in this study were:

H1: Satisfaction is directly related to students’ continued intention.

H2: Confirmation of expectation is directly related to students’ satisfaction.

H3: Perceived usefulness is directly related to students’ satisfaction.

H4: Perceived usefulness is directly related to students’ continued intention.

H5: Attitudes are directly related to students’ continued intention.

H6: Subjective norms are directly related to students’ continued intention.

H7: Perceived behavioral control is directly related to students’ continued intention.

**Table 1.** Definitions of the constructs used in this study

Construct	Definition
Perceived usefulness	Students’ initial expectation of e-learning
Confirmation	Students’ perceived performance and determining the extent to which their expectation is confirmed
Satisfaction	Students’ perceived satisfaction with e-learning
Attitudes	Students’ positive and negative feelings towards e-learning
Subjective norms	The social pressures that influence students’ use of e-learning, such as family and educators
Perceived behavioral control	Students’ beliefs about the ease or difficulty of using e-learning
Intention	Students’ beliefs about expected adoption of e-learning

## Materials and methods

### Data collection

The quantitative approach has been widely used in previous technology research [26,28–30]. For such studies, the questionnaires are considered the most reliable tool to measure relationships among constructs in the research model [28]. In the present study, a questionnaire was sent to the medical and dental students enrolled in seven major universities in Saudi Arabia. The data were collected from students studying from the second to the sixth year. First-year students were excluded because they do not take any medical or dental courses. Students were invited to participate for a period of three months. Participants were given six weeks to complete the online questionnaire. After the initial questionnaire distribution, we sent two reminder emails to increase the response rate during the second and fourth weeks. We used a convenience sampling technique to select the participants. The minimum sample size required for this study was 379, using an estimated prevalence of 50%, a precision level of 5%, and a confidence interval of 90%. However, 1300 invitations were sent, assuming a response rate of 30%.

### Instrument

We used an anonymous close-ended questionnaire to examine students’ satisfaction and continued intention to use e-learning. The questionnaire included two main parts. The first part included personal information like gender, age and year of study. The second part was adapted from the previous questionnaire on which validity evidence has been gathered, and only minor revisions were made according to the context of the present study [19]. It had questions to measure the research model’s constructs such as perceived usefulness, confirmation, satisfaction, attitudes, subjective norms, perceived behavioral control and continued intention. The participants’ responses were measured on a 5-point Likert scale ranging from 1 = Strongly disagree to 5 = Strongly agree. Table 3 represents the constructs and their corresponding items.

**Table 2.** Demographic characteristics

		n	%
Gender	Female	437	50.2
	Male	433	49.8
University	King Abdul-Aziz University	192	22.1
	Taibah University	140	16.1
	Imam Abdulrahman Bin Faisal University	128	14.7
	University of Tabuk	104	12.0
	King Khalid University	84	9.7
	Jazan University	75	8.6
	Umm Al Qura University	58	6.7
	King Saud University	56	6.4
	Najran University	20	2.3
	Alfarabi college	13	1.5
College	Medical college	731	84.0
	Dental college	139	16.0
Year of study	2^nd^ year	93	10.7
	3^rd^ year	159	18.3
	4^th^ year	174	20.0
	5^th^ year	227	26.1
	6^th^ year	217	24.9

**Table 3.** Frequency and Median (IQRa) on satisfaction and continued intention toward e-learning during the period of COVID-19 pandemic (N = 870)

**Construct**	**Measuring items**	**Disagree**	**Neutral**	**Agree**	**Median**	**IQR**a
**Expectation**	Using e-learning can improve my learning performance	228	276	366	2	2
	Using e-learning can increase my learning effectiveness	258	264	348	2	2
	I find e-learning to be useful to me	203	196	471	3	1
**Attitude**	Using e-learning is a good idea	148	207	515	3	1
	I like using e-learning	224	200	446	3	2
	It is desirable to use e-learning	218	265	387	2	2
**Subjective norms**	People important to me support my use of e-learning	123	323	424	2	1
	People who influence me think that I should use e-learning	174	418	278	2	1
	People whose opinion I value prefer that I should use e-learning	182	423	265	2	1
**Perceived behavioural control**	Using e-learning system was entirely within my control	154	210	506	3	1
	I had the resources, knowledge, and ability to use e-learning	67	137	666	3	0
	I would be able to use the e-learning system well for learning process	112	187	571	3	1
**Confirmation**	My experience using the e-learning system was better than I expected	156	204	510	3	1
	The service level provided by the e-learning system was better than I expected	170	244	456	3	1
	The e-learning systems can meet demands in excess of what I required for the service	173	329	368	2	1
**Satisfaction**	I am satisfied with the performance of e-learning	193	253	424	2	1
	I am pleased with the experience of using e-learning	176	250	444	3	1
**Intention to use**	My decision to use e-learning system on a regular basis in the future	254	236	380	2	2
	I will frequently use the e-learning system in the future	199	231	440	3	1
	I will strongly recommend that others use it	190	244	436	3	1

*a1: Strongly disagree and Disagree; 2: Neutral; 3: Strongly agree and Agree; IQR: interquartile range*

### Data analysis

Data were analyzed through descriptive statistical analysis using the statistical software SPSS version 24.0 (IBM Corp. Released 2016. IBM SPSS Statistics for Windows, Version 24.0. Armonk, NY: IBM Corp.). We used structural equation modeling to test the seven hypotheses of this study collectively and provide the path coefficients. Mplus version 7.2 (Muthén and Muthén, 1998–2014) was used to evaluate the measurement model and estimate the structural coefficients.

## Results

### Demographic information

We received a total of 870 completed questionnaires with a response rate of 67%. The mean age of the participants was 22.3 ± 1.9 years. Participants were approximately equally distributed by gender, and the majority were from medical colleges (84%) (Table 2). Participants reported Blackboard (51%) and Zoom (45.6%) as the most commonly used platforms in the university during the pandemic. Approximately two-thirds (65.6%) reported that they received formal training from their university on how to use the e-learning platform. Over half (58.4%) of the participants preferred a combination of e-learning and traditional classrooms after the pandemic. The results for the different constructs are listed in Table 3.

### Hypotheses testing

**Figure 2.**
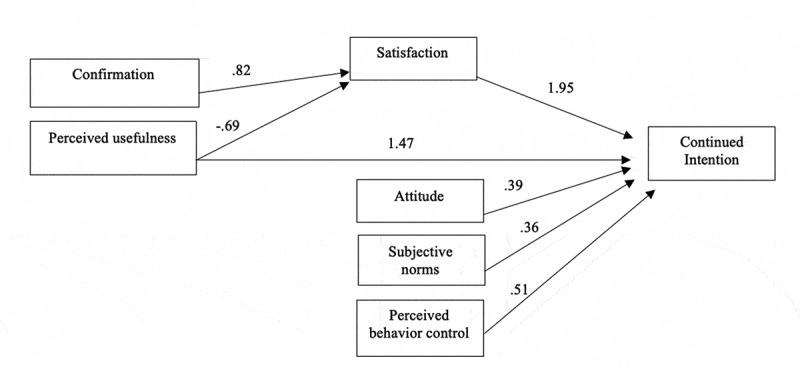
Hypotheses testing results

The path association of each hypothesized association in the research model and variance explained (R2 value) for each path was examined (Figure 2 and Table 4). All associations were significantly associated with p < .05. The intention to re-use e-learning in this study was jointly predicted by perceived behavioral control (β = 0.506, p < .0001), expectation (β = 1.472, p < .0001), and satisfaction (β = 1.945, p < .0001). Satisfaction was negatively associated with expectation (β = −0.959, p < .0001) and confirmation (β = 0.284, p < .0001). These variables together explained 83.7% of the variance of intention to re-use (R^2^ = 0.837).

**Table 4.** Summary of hypothesis tests

Hypothesis			β	p value	Support
Confirmation		Satisfaction	0.284	***	Yes
Expectation		Satisfaction	−0.959	***	Yes
Expectation		Intention to re-use	1.472	***	Yes
Satisfaction		Intention to re-use	1.945	***	Yes
Attitude		Intention to re-use	0.394	***	Yes
Subjective Norms		Intention to re-use	0.359	***	Yes
Perceived behavioral control		Intention to re-use	0.506	***	Yes

*** *p* < .001

## Discussion

Due to the current crisis, there was a sudden shift from traditional lectures to e-learning worldwide to halt the spread of the disease. Although e-learning has the benefit of not having place and time constraints, there are still many challenges. Therefore, it is essential to study students’ attitudes and expectations regarding e-learning. The willingness to adopt e-learning would be considered successfully implemented if students moved from initial adoption to continued usage intention [14,30]. The ECM and TPB guided the study, and their effectiveness in explaining the adoption of new technologies is well documented [20].

The results revealed that our participants were at a moderate level of satisfaction with e-learning. Around half of the participants preferred blended learning. In this regard, our findings are in line with other studies which showed that healthcare students prefer a combination of traditional lectures and e-learning [10]. Today’s students are millennial learners who prefer the integration of innovative teaching modalities and interaction with their instructors to embrace the advantages of both [31]. They prefer complete access to online teaching resources, regardless of time constraints, in addition to communication and the social presence of their instructors. According to Berge, students fear ‘faceless’ teaching and still want face-to-face interaction with their instructors [32]. They also have pedagogical needs that need to be met in their preferred learning styles [31].

Drawing on ECT, perceived usefulness followed by confirmation was the strongest negative predictor of students’ satisfaction. This suggests that students’ belief in the usefulness of e-learning is a determining antecedent of their perception of satisfaction and consequently affects their intention [33]. This is similar to a study which found that medical professionals’ perceived usefulness of a cloud-based learning system significantly influenced their satisfaction, and thereafter affected their continued intention to use it [33]. This finding echoes several studies’ findings which indicate that expectation has a significant effect on users’ satisfaction and intention to use new technologies such as mobile or e-learning [34,35]. Beyond our expectations, however, there was a negative relationship between perceived usefulness and satisfaction. Our finding contradicts other studies as they reported that learners’ initial expectations of using e-learning are a positive predictive factor of their satisfaction [33,36–38]. A possible explanation could be that people’s expectations of a system are part of their previous experience, and healthcare students in Saudi Arabia had no previous experience with e-learning [39].

Next, healthcare students’ confirmation of expectations towards e-learning had a significant positive influence in forming their satisfaction that led to their continued intention to use e-learning. This finding is consistent with Cheng’s results which revealed that medical professionals’ confirmation positively induced their satisfaction with e-learning [33]. Several other studies reported the same findings [20,36,38]. The results suggest that future attempts should be made to prepare students to use e-learning effectively to increase their confirmation of expectations and ensure they are satisfied [19].

As shown in ECT, satisfaction is the major antecedent for predicting students’ continued intention to use e-learning, followed by perceived usefulness. A possible explanation would be that the degree to which students are satisfied with e-learning will affect how they are more likely to continue using it. This finding is in line with the results of Chou et al., revealing that patient satisfaction was a core factor in determining continued intention to use e-learning as a health education tool [20]. In another study, Shiue et al. evaluated mobile learning to teach students the right medication used for health education and evaluated their continuance usage intention [40]. Their results suggested that students’ satisfaction with mobile learning was a key predictor of students’ continuous intention. From marketing literature, researchers claim that customers with a high level of satisfaction are more likely to use a system than less satisfied customers [41]. In an educational context, we can construct a similar argument to ensure that students continue using e-learning; it is essential to improve reliability of satisfaction.

Apart from the above discussion, our findings showed that perceived usefulness can directly influence students’ intention to use e-learning. These findings support those of previous studies [38,42–44]. If students find e-learning useful, beneficial, easy to use, and improve their learning experience, their likelihood of continuing to use e-learning in the educational process will increase.

TPB is an alternative theory to perceive the intention to use a technology [45]. Imposing this theory, the constructs of attitudes, subjective norms, and perceived behavioral control had significantly influenced students’ e-learning intention. Among the three constructs, perceived behavioral control and attitudes had higher effects, followed by subjective norms. Our result is similar to prior research which implied that e-learning usage intention is dependent on individual behavior control [23,28]. Another study reported that medical students’ behavioral control was a determining factor in their intention to continue receiving m-learning [24]. Hadadgar et al. also showed that general practitioners’ behavioral control played a significant role in predicting their intention to use e-learning in continued medical education [28]. This indicates that empowering students with high controllability and self-confidence would increase the likelihood of students’ acceptance of mandatory e-learning.

The results demonstrated that students’ attitudes play a pivotal role in determining their intention. This finding agrees with that of Hadadgar et al. [28], Chu et al. [46], Cheon et al. [23], Raza et al. [47]. Derived from the theory of planned behavior, attitude is a decisive factor that implies that learners’ positive or negative feelings about technology would affect their intent. Hence, healthcare students would consider continuing using e-learning if they had positive attitudes and feelings toward the system. Educators should be aware of the students’ psychological aspects and reduce their psychological resistance to e-learning. Finally, a significant relation between subjective norms and e-learning intention was noted. Nonetheless, this impact was lower than that of the other two constructs. Our finding is congruent with those of previous studies, which reported that subjective norms weakly affected the use of technology [23,45]. However, this result was not in line with other studies that demonstrated that subjective norms affect mobile or e-learning intention [24,26]. Our result reflects healthcare students need their colleagues’ and instructors’ support to increase their willingness to continue e-learning during the pandemic and in the future.

## Implications and limitations

Theoretically, the present study adds to the existing literature on e-learning by integrating two well-known theories, ECT and TPB. From the above discussion, all the proposed hypotheses in the present study were supported. Therefore, this study provides insights for educators and academic institutions on retaining students’ e-learning satisfaction and intention during the pandemic. According to the results, to drive students’ satisfaction with e-learning, decision-makers in universities should consider providing their students with an easy, useful, and high-quality e-learning platform. Hence, academics should adopt easy-to-use platforms that increase students’ confidence to meet their expectations with minimal problems. The more satisfied students, the more their intent to continue using e-learning will be. In addition, due to the unexpected sudden move to e-learning, academic institutions should encourage students’ perceived behavioral control and improve their attitudes toward e-learning by providing orientation and training on their universities’ different platforms. This will boost their self-confidence and experience, which will enhance learning outcomes. Finally, it is worth mentioning that peers and educators influence students; therefore, instructors need to support and motivate their students to actively engage in the teaching process during the pandemic.

There are some limitations in this study. First, we did not consider the emotional aspect. During the outbreak, quarantine and social isolation were measures to reduce the spread of the disease. This could be stressful and overwhelming for students and could affect their emotional state. Second, we did not look if demographic variables were predictors of students’ satisfaction and intention. We suggest that future studies should investigate their effect as they might provide additional insights. Moreover, our analyses may be subject to a non-response bias that may account for samples that is not representational of certain demographic groups. One way that this nonresponsive bias may be manifested is that those who have no issues with e-learning may not have had any interest in taking the survey. Finally, we did not study instructors’ perspectives. Due to the sudden sweep to online teaching, there was not enough time to prepare for e-learning. Some academics were not familiar with e-learning; hence, their views are impactful. Future studies are needed to analyze faculty members’ satisfaction and intention.

## Conclusion

The COVID-19 pandemic has dramatically changed the educational system around the world. As a consequence, post-pandemic pedagogy might change, and universities may continue online teaching. E-learning might be the future of education and the key to improving certain pedagogical approaches such as self-directed and lifelong learning. Therefore, this study will guide academics on how to focus their efforts to consider integrating e-learning into the healthcare curriculum.

## Data Availability

All data are available from the corresponding author upon request.
